# Predictive modeling for COVID-19 readmission risk using machine learning algorithms

**DOI:** 10.1186/s12911-022-01880-z

**Published:** 2022-05-20

**Authors:** Mostafa Shanbehzadeh, Azita Yazdani, Mohsen Shafiee, Hadi Kazemi-Arpanahi

**Affiliations:** 1 Department of Health Information Technology, School of Paramedical, Ilam University of Medical Sciences, Ilam, Iran; 2 Clinical Education Research Center, Health Human Resources Research Center, Department of Health Information Management, School of Health Management and Information Sciences, Shiraz University of Medical Sciences, Shiraz, Iran; 3 Department of Nursing, Abadan University of Medical Sciences, Abadan, Iran; 4 Department of Health Information Technology, Abadan University of Medical Sciences, Abadan, Iran; 5 Department of Student Research Committee, Abadan University of Medical Sciences, Abadan, Iran

**Keywords:** COVID-19, Machine learning, Data mining, Patient readmission

## Abstract

**Introduction:**

The COVID-19 pandemic overwhelmed healthcare systems with severe shortages in hospital resources such as ICU beds, specialized doctors, and respiratory ventilators. In this situation, reducing COVID-19 readmissions could potentially maintain hospital capacity. By employing machine learning (ML), we can predict the likelihood of COVID-19 readmission risk, which can assist in the optimal allocation of restricted resources to seriously ill patients.

**Methods:**

In this retrospective single-center study, the data of 1225 COVID-19 patients discharged between January 9, 2020, and October 20, 2021 were analyzed. First, the most important predictors were selected using the horse herd optimization algorithms. Then, three classical ML algorithms, including decision tree, support vector machine, and k-nearest neighbors, and a hybrid algorithm, namely water wave optimization (WWO) as a precise metaheuristic evolutionary algorithm combined with a neural network were used to construct predictive models for COVID-19 readmission. Finally, the performance of prediction models was measured, and the best-performing one was identified.

**Results:**

The ML algorithms were trained using 17 validated features. Among the four selected ML algorithms, the WWO had the best average performance in tenfold cross-validation (accuracy: 0.9705, precision: 0.9729, recall: 0.9869, specificity: 0.9259, F-measure: 0.9795).

**Conclusions:**

Our findings show that the WWO algorithm predicts the risk of readmission of COVID-19 patients more accurately than other ML algorithms. The models developed herein can inform frontline clinicians and healthcare policymakers to manage and optimally allocate limited hospital resources to seriously ill COVID-19 patients.

## Introduction

The coronavirus disease 2019 (COVID-19) or acute respiratory syndrome coronavirus 2 (SARS-CoV-2) is a highly transmissible and widespread infection that, in its severe form, causes serious damage to the respiratory tract and in some individuals leads to pneumonia, multi-organ failure (MOF), and even death [1, 2]. The unknown clinical course and behavior of COVID-19 contributed to ambiguous discharge criteria for hospitalized patients [3]**.** Furthermore, the variability and dynamic nature of the virus and its new variants led to resistance to treatment and vaccinations [4–6]. According to reports, about 5% of definitive COVID-19 cases require hospitalization care services, and the rate of hospital readmission due to this disease varies from 2 and 10% in different studies [7, 8]. This rate varies depending on age, body mass index, underlying diseases, sex, vaccination, disease severity, and SARS-CoV-2 (COVID-19) variant types (Alpha, Beta, Delta, Omicron) [9–11]. After second-and third-dose vaccination, this rate considerably decreased [12].

Hospital readmission is defined as the admission of a patient to a hospital at a specific time within 30 to 60 days after discharge from the hospital. Readmissions represent important and costly events that impose a heavy burden on patients’ families and the healthcare systems [13, 14]. Hospital readmissions are mostly accountable for the reputation of the healthcare settings, causing notoriety and indicating clinicians' carelessness [15]. Hospital readmission has received increasing attention as the main performance indicator for evaluating the quality of care given to patients [16, 17]. Studies report that over 60% of hospital readmissions are potentially preventable. However, due to the varied and complex natures of factors causing disease recurrence and readmissions, caregivers cannot process all the information to precisely detect endangered patients [18]. Thus, increasing attention is being paid in the scientific community to this problem from a data analysis viewpoint [19].

Hospital readmission is known as a key indicator of the quality of service during the COVID-19 pandemic [20]. As the prevalence of COVID-19 increased and many communities became severely impacted, the healthcare systems of many countries failed to meet the growing needs of patients [21]. Many patients in such conditions were discharged after admission with relative recovery. Meanwhile, due to the unknown and aggressive nature of the disease, the readmission rate of patients increased [22, 23]. Readmission imposes additional costs on healthcare organizations and patients [22]. It also reduces the quality indicators of service delivery and raises the rate of serious complications and death during the pandemic [24, 25].

The use of clinical evaluation methods to predict disease re-infection and readmission is usually expensive, difficult, and lacks optimal predictive accuracy as it does not use cumulative patient data [26]. Scoring indices and conventional statistical models can only analyze simple and linear relationships between variables. Nevertheless, the unknown and multidimensional nature of COVID-19 requires innovative technologies such as artificial intelligence (AI) to analyze the nonlinear and complex relationships between variables [26–35]. Machine learning (ML), which is a major branch of AI, reveals new and practical patterns from huge raw datasets [36, 37]. ML algorithms diminish uncertainties and ambiguities related to new diseases such as COVID-19 by providing diagnostic and predictive models based on valid and scientific evidence [38, 39]. The multifaceted interaction between readmission and possible risk factors makes the precise prediction of readmission difficult. ML approaches can deal with high-dimensional clinical data to produce precise patient risk stratification models and shape healthcare decisions through the customization of care [36, 39].

Numerous studies have examined the application of ML and deep learning (DL) methods to predict the disease recurrence, reinfection, and patient deterioration among recovered COVID‐19 patients [40–44]. ML methods are more accurate than conventional statistical models for predicting hospital readmission in COVID-19 hospitalized patients [45–47]. Therefore, this study aimed to apply ML algorithms to predict the likelihood of hospital readmission of COVID-19 patients. The current study sought to answer two questions: What are the most important predictor variables affecting the readmission of COVID-19 patients? and Which ML model is more effective for predicting readmission in these patients?

## Materials and methods

### Study design

The current research was a retrospective study on the data of 2854 patients discharged from a 400-bed academic hospital in Abadan, Iran, from January 9, 2020 to October 20, 2021. The patient data were extracted from the COVID-19 hospital-based registry database. The implemented registry system is a comprehensive web-based application software that records patient data for clinical and research purposes in five main sections: demographic, diagnostic and therapeutic, paraclinical, and history and information. Patients aged less than 18 years, those who were admitted for non-COVID-19 conditions, died during hospitalization, were discharged against medical advice, or had incomplete case records with > 70% missing data were excluded from the study.

The study was conducted in three phases. In the first phase, the primary raw dataset was preprocessed. In the second phase, important features for predicting the risk of hospital readmission in COVID-19 patients were selected using meta-heuristic algorithms (MHAs). After identifying the most important features, three traditional ML algorithms and a meta-heuristic algorithm for water wave optimization using a neural network were trained. Finally, the developed models’ performances were compared, and the best algorithm was determined. The study protocol was approved by the Abadan University of Medical Science Ethics Board (ABADANUMS.REC.1400.136),https://ethics.research.ac.ir/ProposalCertificateEn.php?id=246118&Print=true&NoPrintHeader=true&NoPrintFooter=true&NoPrintPageBorder=true&LetterPrint=true).

### Data preparation

We clustered certain classes to decrease the number of classes of these variables. Records with more than 70% of missing data were excluded from the analysis. For the remaining missing values, presuming that the missing data were distributed randomly, the imputation technique which is a common method to deal with missing values was adopted [19]. To manage noisy data, the normal range of each variable was first defined using the opinion of two infectious diseases specialists, a virology expert, and a hematology expert. Then, we specified all the values that were outside the defined range and filled them by referring to patient records or the responsible doctor. Because the p-value cut-off point was < 0.05 in this study, the median substitution was used instead of the mean for the missing values. In other words, we did not fill them with the mean values due to the uneven distribution of variables.

### Data balancing

A major barrier to the use of ML algorithms is the problem of imbalanced data, which happens when classes are not categorized equally. In the selected dataset, the amount of data in outcome classes is significantly imbalanced and contains more samples related to the non-readmission class (1136 cases), while the readmission class is much smaller (only 89 cases). Accordingly, the developed models often deliver biased results towards the overriding class, and the ML models are much more likely to categorize new observations into the majority class. Herein, to handle class imbalance, the synthetic minority over-sampling technique (SMOTE) was employed in the Imbalanced-Learn toolbox to balance the dataset. We performed a Kolmogorov–Smirnov statistical test to check the normality and skewness of the data, the results of which showed that the data followed a normal distribution.

### Predictor and outcome variables

#### Predictor variables

The data for analysis included six categories of predictor variables extracted from the hospital’s COVID-19 dataset. Sixty variables were categorized as demographic characteristics (six variables), clinical manifestation (14 variables), medical history and comorbidities (eight variables), laboratory results (28 variables), treatment (one variable), and radiological indicators (two variables).

#### Outcome variable

It calculated whether the patient was readmitted on the last visit within 30 days after being discharged from the hospital on the penultimate visit (coded 1) or not (coded 0). The detailed descriptions of all the variables are listed in Table 1.

**Table 1:** A list of variables and their corresponding category utilized in predicting COVID-19 readmission risk

Type	Category	Variables
Inputs	Demographic characteristics	Age, sex, height, weight, blood group, hospitalization length of stay (LOS)
	Clinical manifestation	Dry cough, nausea, headache, gastrointestinal (GI) manifestation, Chill, loss of taste and smell, rhinorrhea, sore throat, contusion, high body temperature, muscular pain, vomiting, dyspnea
	Past medical history and comorbidities	Cardiac disease, smoking, pneumonia, hypertension (diastolic/ systolic), alcohol addiction, diabetes, and other underline diseases
	Laboratory results	Red-cell count, hematocrit, hemoglobin, absolute lymphocyte count, blood calcium, blood potassium, absolute neutrophil count, alanine aminotransferase (ALT), magnesium, prothrombin time, alkaline phosphatase, platelet count, hypersensitive troponin creatinine, white cell count, aspartate aminotransferase (ASP), blood glucose, total bilirubin, erythrocyte sedimentation rate (ESR), C-reactive protein(CRP), albumin, thromboplastin time, lactate dehydrogenase (LDH), D-dimer, blood phosphorus, blood sodium, and blood urea nitrogen (BUN), oxygen saturation
	Radiological factors	Pleural fluid, consolidation
	Treatment	Oxygen therapy
Output	Readmission: yes (1), no (0)	

### Feature selection

Feature selection can be performed to enhance the prediction precision and reduce the algorithm's run time by selecting the most important variables, thereby alleviating the model’s computational intricacy [48]. In this study, the efficiency of several feature selection methods was compared to identify the best predictors. To this end, six well-known MHAs, including horse herd optimization algorithm (HOA), particle swarm optimization (PSO), genetic algorithm (GA), grey wolf optimization (GWO), differential evolution (DE), and Harris hawks optimization (HHO) were utilized for feature selection. In this phase, all the experiments were carried out using MATLAB 2019. To evaluate the performance of MHAs in identifying the most effective factors, three performance evaluation metrics of the mean fitness value, classification accuracy using k-nearest neighbors (KNN), and the number of selected features were calculated.

### Model development

We trained four ML algorithms, namely KNN, water wave optimization (WWO), support vector machine (SVM), and decision tree (DT) in the WEKA application. Each method is described below.

#### SVM

The SVM is a supervised algorithm associated with datasets having data class labels. This algorithm can detect the pattern and assign the sample to specified output classes. With a high dimension of dataset, this algorithm has a proper classification potential. Contrary to artificial neural networks (ANNs), it is not stopped at the local maximum during the training process. This algorithm focuses on the line discriminating various class labels with high capability when there are complicated databases and patterns and enhancing the line. Generally, the SVM aims to find the hyperplane in categorizing the dataset sample to obtain the best classification performance in n-dimensional datasets. This capability of SVM contributes to its good performance compared to other approaches [49–51].

#### KNN

This algorithm, similar to the SVM algorithm, can be used for classification and regression. It is a supervised ML algorithm when considering an output class for the dataset. For a specific value of K, an object belongs to the classes according to its nearest samples. This algorithm does not need to assume the data pattern before classifying the objects. The KNN is classified as a lazy algorithm because the learning process is not concurrent with the algorithm training. In the training process, the data are stored and will be categorized when training the new data instances. Some advantages of this algorithm include its lack of training time because of being lazy, simple implementation with specified K and Euclidean distance, lost value imputing, and excellent performance thanks to its independence from new data instances [52–54].

#### DT

Decision trees are ML algorithms and have a potential structure for induction and interpretation in the ML process. This algorithm consists of three node types in their structural tress: roots, internal nodes, and external nodes named leaves. The root node in DT belongs to the dataset attribute with high capability in discriminating the output classes, i.e., the most crucial variable in the study. The internal nodes link the root to external nodes in trees; therefore, this structure can trace the tree from the root to leaves mediated by internal nodes to obtain the IF–THEN rules. The external nodes or leaves are places where the samples can be classified. In reality, the number of leaves constitutes the number of induction rules extracted from the tree. The benefits of this induction structure include simplicity for interpretation, easy implementation because of less complicated calculations, and less need for data normalization [55–58].

#### Proposed method

In this study, using a meta-heuristic algorithm for optimizing water waves, a model is presented for predicting the risk of readmission of COVID-19 patients. In the proposed model, the novel WWO algorithm was adopted to minimize the classification error. This algorithm cannot make predictions alone, so it is combined with the ANN algorithm. In other words, the proposed model uses the WWO evolutionary algorithm to promote the accuracy and effectiveness of predicting the readmission risk of COVID-19 patients. In optimization problems, modeling natural and biological phenomena is an effective method. This algorithm uses the existing relationships between water waves and their feedback to the environment to solve optimization problems. In the WWO algorithm, like any metaheuristic or evolutionary algorithm, sets of initial solutions are encoded in the form of a population. In this meta-heuristic algorithm, each problem solution is identified as a wave, and sets of waves are considered as the initial population of the problem. In a WWO algorithm, each solution to a problem or wave is encoded with properties such as wave height or wavelength. In the WWO algorithm, the solutions to the problem are first encoded as waves and several waves are randomly scattered in the problem search space.

**Fig. 1:**
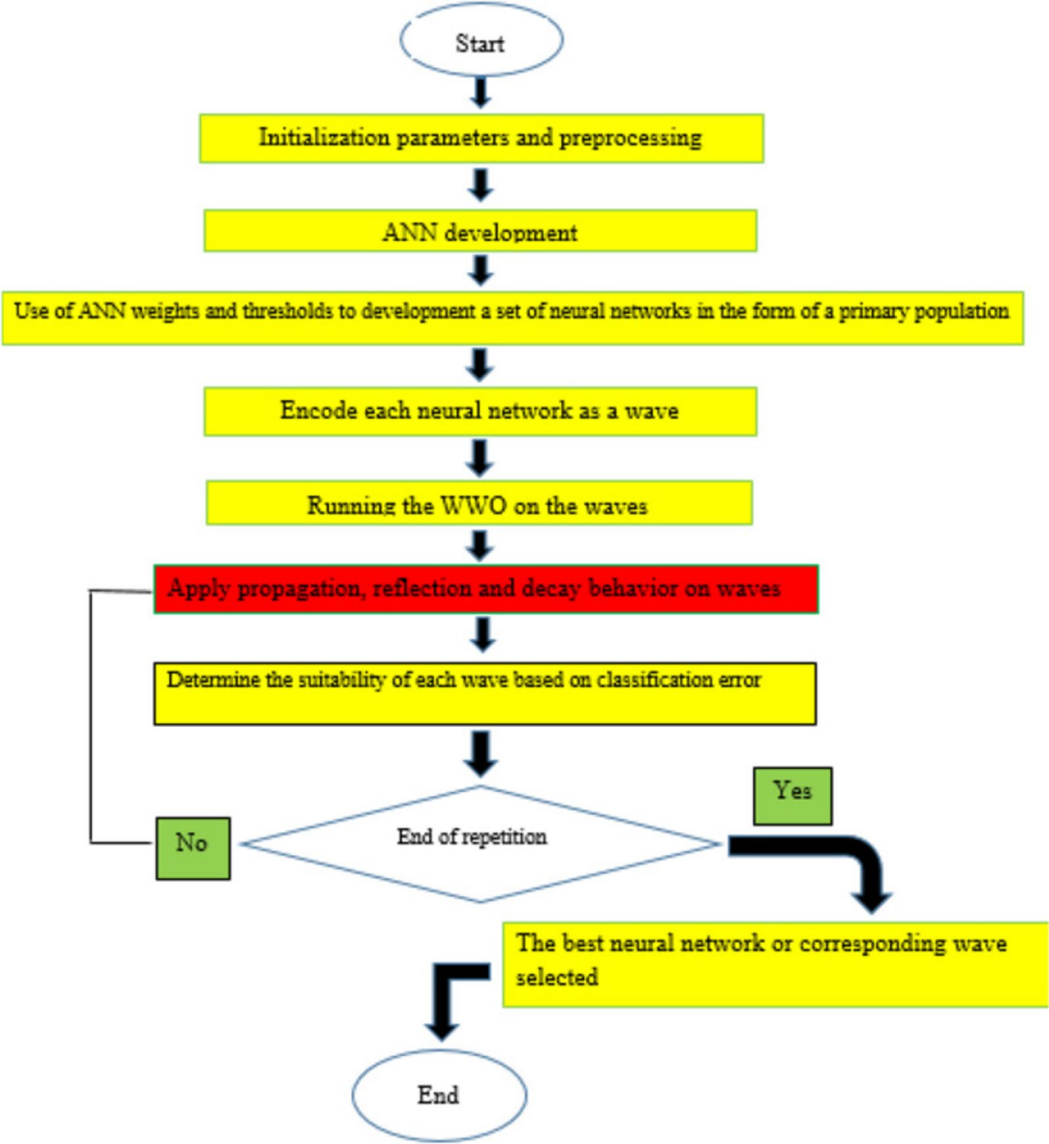
The proposed method framework for predicting the risk of COVID-19 readmission

In the proposed framework, a multilayer neural network is first created based on the training data set. Subsequently, the desired ANN is created as an array of weights and thresholds under the initial population of water waves. Afterward, a WWO algorithm is implemented on them to finally develop the best water wave or the corresponding ANN to predict the risk of readmission of COVID-19 patients. A multi-layered neural network with two hidden layers and five hidden nodes in each layer is randomly selected for initial training by 70% of the entire data. The desired ANN configuration is optimized by the WWO algorithm and implemented in MATLAB R2016a to select the best member of the neural network set. The performance of the proposed model was compared with other methods. To calculate the average error in the experiments, the number of experiments was considered to be 50, and the mean error in all these experiments was announced as the final result. Mean square error (MSE) and root mean square error (RMSE) were used as the objective function to reduce the error. In 50 experiments, values of 0.17 and 0.41 were respectively calculated. In the proposed method, an ANN is initially created by training, and several neural networks are encoded in the form of water waves. The waves are optimized, and then each of these waves (corresponding neural network) is evaluated by the objective function of the problem, and the best water wave or neural network is identified in this iteration of the algorithm. Any ANN or water wave that has a smaller classification error is considered to be better qualified. Figure 1 describes the steps of the proposed model.

### Models evaluation

To evaluate the performance of each algorithm, tenfold cross-validation was used to obtain reliable results for assessing prediction models or obtaining reliable results. The original training dataset was divided into 10 folds through stratified random sampling. For the *i*th iteration, fold *i* was considered as the test data, and the remaining nine folds were used to train the model. The model was assessed using the test data, and the procedure was repeated for 10 iterations. The evaluation results of 10 iterations were collected to compute the mean value and standard deviation.

The performance of models was measured using accuracy, precision, recall, specificity, and F-measure metrics. These evaluation criteria are commonly reported in the evaluation of models with ML [59], and their definitions are listed in Table 2. Furthermore, Friedman's statistical technique was adopted to compare the algorithms more precisely and select the algorithm with the highest efficiency. This test assigns a rank to each algorithm and the best algorithm has a lower rating. The null hypothesis states that all the algorithms are the same, while rejecting the null hypothesis shows that the compared algorithms significantly differ. In this paper, we set the significance level to α = 0.05.

**Table 2:** Definitions of evaluation metrics

Performance measures	Definitions
Precision	TP/(TP + FP)
Specificity/true negative rate (TNR)	TN/(TN + FP)
Sensitivity/true positive rate (TPR) or Recall	TP/(TP + FN)
Accuracy	(TP + TN)/(TP + TN + FP + FN)
F-measure	(2 × Precision × Recall)/ (Precision + Recall)

^*^True positive (TP), true negative (TN), false positive (FP), false negative (FN)

## Results

### Sample characteristics

After applying the exclusion criteria, the records of 1225 discharged COVID-19 patients remained. Of these, 887 (72.40%) were male and 338 (27.60%) were women, and the median age of the participants was 57.25 years (interquartile 18–100). Of these, 89 patients had readmission, and 1136 patients had no readmission.

### Feature selection

Given that MHAs are naturally random and the solutions may be slightly different in each independent execution, each algorithm was executed 20 times, and the average of the results was obtained after 20 independent executions. Furthermore, in all algorithms, the population size and the maximum number of iterations were set to 50 and 100, respectively. The mean fitness value of each algorithm, the accuracy of the KNN classifier based on the selected features, and the number of selected features are presented in Table 3.

**Table 3:** Comparison of algorithms in terms of different criteria in 20 runs

Measure	Algorithms					
	GA	PSO	DE	GWO	HHO	HOA
Mean fitness value	0.101 (95% CI 0.103 to 0.099)	0.095 (95% CI 0.096 to 0.094)	0.096 95% CI 0.095 to 0.097)	0.098 (95% CI 0.099 to 0.097)	0.101 (95% CI 0.102 to 0.099)	0.083 (95% CI 0.082 to 0.084)
Accuracy	0.891 (95% CI 0.888 to 0.894)	0.903 (95% CI 0.901 to 0.905)	0.904 (95% CI 0.903 to 0.905)	0.900 (95% CI 0.901 to 0.899)	0.892 (95% CI 0.891 to 0.893)	0.924 (95% CI 0.923 to 0.925)
No. selected features	18	21	20	24	19	17

The numerical results show that the HOA algorithm is significantly superior to the other algorithms in terms of all three criteria [accuracy: 0.924 (95% CI 0.923 to 0.925)]. The most important variables to predict the readmission rate selected by HOA were age, sex, prior LOS, fever, dry coughs, cardiovascular disease, diabetes, hypertension, prior oxygen therapy, CRP, creatinine, ESR, D-dimer, ALT/ASP, absolute lymphocyte/ neutrophil count, pleural effusion and consolidation.

### Model implementation

To select the best predictive performance, three traditional ML algorithms and a hybrid technique were trained, and their performance was compared according to the selected evaluation criteria. The steps of the proposed method (hybrid) for predicting the readmission risk of COVID-19 patients are as follows:

First, a multilayer artificial neural network with a specified number of hidden layers was trained by the COVID-19 dataset. Next, by training the desired ANN, the values and biases of the multilayer neural network were quantified, so several multilayer ANNs were developed with the same weights and thresholds and with relatively different values. Then, each of these neural networks created by the proposed coding was converted into several arrays or water waves, which constituted the initial population quantification step in the WWO algorithm. Each of the water waves or the initial population of the corresponding ANNs was delivered as an input to the wave optimization algorithm; then, each wave (the corresponding neural network) was evaluated by the objective function of the problem and the best water wave or the same neural network was detected in this iteration. The WWO algorithm was implemented on neural networks or water waves to extract the best wave or neural network to predict the re-admission risk in the last iteration. Finally, the efficiency of the proposed method was assessed based on model evaluation criteria.

Note that the performance of ML models on the initial dataset as well as the dataset after feature selection was implemented (trained) and compared separately (see Table 4).

**Table 4:** The performance of ML algorithms before and after preprocessing

ML algorithm	Accuracy		Precision		Recall		Specificity		F-Measure		F.a. r	p-value
	b.p	a.p	b.p	a.p	b.p	a.p	b.p	a.p	b.p	a.p		
Decision tree	0.761	0.958	0.564	0.961	0.534	0.903	0.906	0.982	0.547	0.922	2.04	0.0091
SVM	0.457	0.821	0.287	0.743	0.412	0.792	0.375	0.921	0.336	0.767	4	0.0001
KNN	0.526	0.941	0.462	0.942	0.485	0.765	0.912	0.961	0.471	0.823	2.201	0.0063
Proposed model	0.782	0.9705	0.8064	0.9729	0.8333	0.9863	0.7	0.9259	0.8196	0.9795	2.187	0.0065

*b.p* Before preprocessing, *a.p* after preprocessing, *F.a. r* Friedman aligned ranks

Generally, the results in Table 4 reveal that the performance of ML algorithms in the prediction of readmission has improved significantly after preprocessing. The WWO classifier was introduced as the best algorithm for predicting the readmission risk of COVID-19 patients with a 0.9705 accuracy, 0.9729 precision, 0.9869 recall, 0.9259 specificity, and 0.9795 F-measure. The SVM with accuracy, precision, recall, specificity, and F-measure of 0.821, 0.743, 0.792, 0.921, and 0.767 had the poorest performance, respectively.

Given that the data in the outcome classes are unevenly distributed, the F1 score criterion is a more appropriate indicator than accuracy for model evaluation. Herein, due to the imbalance of readmission and non-readmission classes, according to Table 4, the F1 score criterion related to the proposed model was evaluated. With a value of 0.9795, the F2 index indicated the appropriate performance of the proposed model compared to other ML algorithms.

**Fig. 2:**
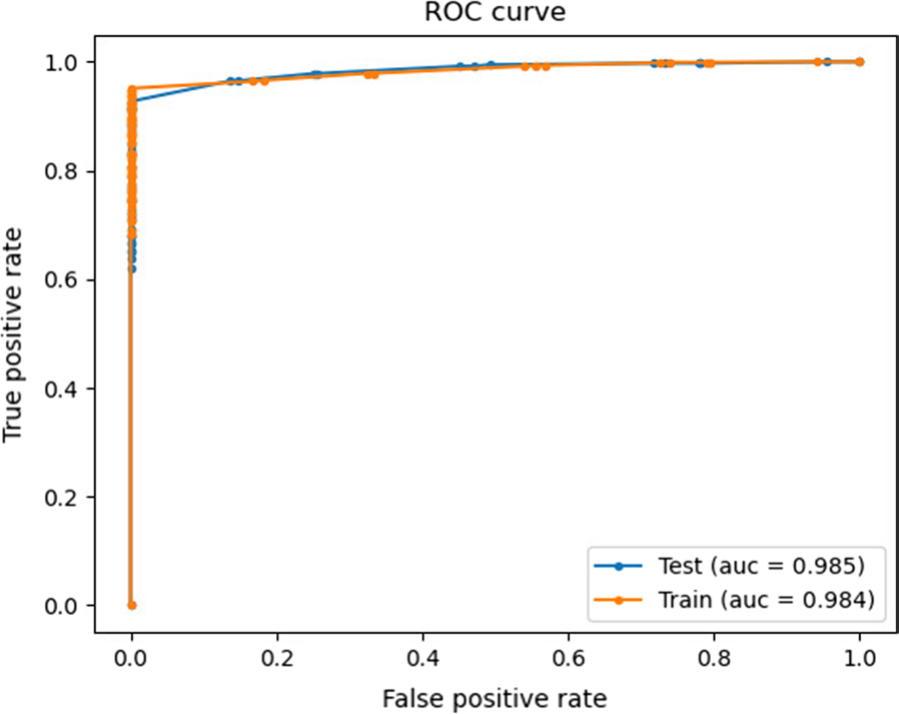
ROC curve for the proposed model

AUC is an effective technique to summarize the accuracy of predictive models. Its value ranges from 0 to 1, with the value of 0 indicating a completely incorrect test and 1 denoting a completely accurate diagnostic test. In general, an AUC of 0.5 does not indicate any discrimination, 0.7 to 0.8 is considered acceptable, 0.8 to 0.9 is considered excellent, and > 0.9 is regarded as prominent [60]. According to Fig. 2, the ACU of the proposed model in the test dataset was excellent.

## Discussion

Accurately identifying the COVID-19 readmission risk can provide a practical solution for clinical decision-making to prevent disease reinfection and recurrent [31]. The present study retrospectively identified the most contributing factors in predicting the risk of hospital readmission in COVID-19 patients. The most important clinical variables were first selected and then leveraged as inputs for constructing ML models including KNN, SVM, WWO, and DT. Finally, the efficiency and performance of developed models were evaluated and compared.

Improving the quality of healthcare services and optimal management of hospital resources has given rise to the need to design predictive models to predict future disease behaviour and outcome [9, 10]. Using decision support systems to predict patient readmission and disease recurrence plays a crucial role in improving care quality and safety [26, 32]. The need to reduce the costs of early readmission up to 30 days after discharge and promote satisfaction during the pandemic has attracted the attention of many researchers [61].

Many studies on ML application to predict readmission have focused on chronic conditions such as cardiovascular diseases [62–68], stroke [69–73], and respiratory diseases [74–78]. Shang (2021) [79], Vosough (2021) [80], and Lin (2019) [81] assessed the performance of ML algorithms in disease recurrence and readmission prediction. Their results showed that ML methods provide a reasonable level of accuracy and certainty in predicting hospital readmission for chronic patients.

Several efforts are also made to apply ML algorithms for the prediction of readmission risk of COVID-19 patients. Mejia et al. concluded that the lack of a valid and scientific model for predicting readmission of COVID-19 patients influences the higher mortality due to disease recurrence [82]. Afrash et al. suggested the ML-based predictive models as useful for managing limited healthcare resources during the COVID-19 pandemic [83]. Donnely et al. also stated that the prediction of COVID-19 readmission is a challenging but important task in preventing the devastating effects of disease recurrence or reinfection [22]. Gavin et al. presented a predictive model to predict 30-day readmission in COVID-19 patients based on the simplified hospital score method for reducing patient readmission and directing resources toward high-risk cases [84]. Hebert et al., developed a risk score model for early prediction of the hospital readmission risk using multiple logistic regression techniques [85]. Rodriguez et al. also proposed a predictive model for readmission of COVID-19 patients based on statistical regression techniques with an AUC-ROC of 0.871 [86].

Eckert et al. reported that predictive modeling for patient readmission based on ML methods can identify high-risk groups of patients with high accuracy; in this way, unplanned readmission and severe complications of the disease will be reduced [87].

Accordingly, Cuong et al. concluded that ML techniques had a greater ability to predict patient readmission during COVID-19 than traditional statistical methods [88]. Davazdahemami et al. used the ML method to predict early or emergency readmission (less than 7 days) in COVID-19 patients. Their proposed model with an AUC of 0.883 showed good performance [33]. Raftarai et al. compared the performance of selected ML algorithms for predicting readmission among COVID-19 hospitalized patients [32]. Jia et al. also assessed the performance of some ML algorithms to predict future deterioration and readmission risk among discharged patients with COVID-19 [89]. Koteswari et al. utilized ML techniques to predict the readmission probability of various COVID-19 cases [15]. In other studies by Ryu [90] Zhao [91], Darabi [92], Chen [93], and Shah [94], ML algorithms were applied to predict the likelihood of readmission of COVID-19 patients.

In our study, the results showed that the WWO algorithm with an accuracy of 0.9705, precision of 0.9729, recall of 0.9869, specificity of 0.9259, and F-measure of 0.9795 has the best capability for early prediction of the risk of readmission in discharged COVID-19 patients.

Selecting key variables affecting the COVID-19 readmission is critical to developing predictive models [9]. Using these variables as an input to ML models improves their performance [32]. Thus far, several studies have selected clinically important predictors for post-discharge COVID-19 recurrence and readmission risk. In Rodriguez's study, some variables (e.g., LDH, CRP, and ESR) were selected as the key factors in hospital readmission [86]. Mendito et al. also determined a number of clinical characteristics such as age, neutrophilia count, sequential organ failure assessment (SOFA), LDH, CRP, and D-dimer as highly contributing factors to the readmission of COVID-19 patients [95]. In the study by Duarte et al., polymerization, living in residential care homes, general malaise, thoracic pain, and hematologic symptoms along with headaches, depressive symptoms, nephrological manifestations, syncope or hypotension, and superinfection were selected as the most relevant factors in COVID-19 readmission [96]. In many studies, age, sex, BMI, length of stay (LOS), ICU hospitalization, and the presence of comorbidities were introduced as the most influencing factors on COVID-19 readmission [97]. In the study by Nematshahi et al., the increase in the time interval from discharge to readmission, age (over 60 years), sex (male), diabetes, elevated creatinine, and lung involvement were selected as influential factors in predicting the readmission of COVID-19 patients [98]. Similarly, in Jeon's research, age and sex were effective in increasing the risk of readmission of COVID-19 patients [99]. The presence of comorbidities, high BMI, adult age, and laboratory indicators such as CRP, creatinine, and ALT/ASP rate were also introduced as the major underlying factors for readmission in COVID-19 patients in Verna's study [100]. In a systematic review conducted by Akbari et al., it was concluded that male sex, white ethnicity, comorbid diseases, and old age affect COVID-19 readmission [101].

In our study, after comparing the performance of six MHAs for feature selection, the HOA method with a mean fitness value of 0.083 and a KNN accuracy of 0.924 achieved the best performance. A total of 17 highly correlated variables such as old age, high weight, dry coughs, fever, dyspnea, loss of smell, cardiovascular diseases, hypertension, CRP, ALT/ASP, SPO2, and leukocytosis were selected as the top predictors affecting COVID-19 readmission.

The proposed model can help healthcare providers in the timely detection of patient deterioration in order to reduce severe complications and the resulting mortalities. Although the current study presented an optimum performance in predicting the readmission risk of patients with COVID-19, it had several potential limitations and challenges. This was a retrospective and single-center dataset, which might have affected the quality, comprehensiveness, and generalizability of the data. In this situation, the existence of some non-integrated, incomplete, error-prone, and abnormal data fields could have negatively impacted prediction. Therefore, to improve the consistency of data, the normal range of each variable was defined using the opinion of two infectious diseases specialists, a virologist, and a hematologist. Then, all the values that were outside the defined range (noisy fields) were specified and completed by referring to patient records or the responsible physician. In addition, the records with more than 70% of empty fields were removed and imputed by median and mode values substitution for continuous and discrete variables, respectively. Moreover, we used only four (albeit well-known) ML algorithms for prediction analyses based on some clinical features. The accuracy and generalizability of our models can be enhanced if other ML techniques are tested on a larger, multicenter, and prospective dataset containing time-varying covariates to identify a more insightful set of longitudinal factors related to COVID-19 readmission. Besides, the external validation method should be used to confirm the results of the present study. Another possible limitation was that this study did not describe any causal relationship between the predictor and outcome variables. This was not the main purpose of this research, but it can be addressed in future studies. Overall, the integrity of predictive models based on ML algorithms depends on the comprehensiveness of the dataset. Since all analyses were based on a single-center dataset, the results of this study may not be generalizable enough for national use. In future research, by analyzing data from multiple COVID-19 care centers in different provinces of Iran, the comprehensiveness and generalizability of the proposed model can be improved.

## Conclusions

Our models have a satisfactory potential in equipping physicians and healthcare policymakers with a practical and effective tool for the timely prediction of hospital readmission of COVID-19 patients. The insights provided by these predictive models may help better care delivery, lessen clinicians’ workload, and ultimately enhance both care quality and financial outcomes. In the present study, the proposed hybrid WWO algorithm yielded the best capability to predict COVID-19 hospital readmission based on influential features. In future studies, the proposed method can be applied to predict the risk of hospital readmissions for other chronic diseases. The MHA used in feature selection can also be improved.

## Abbreviations

MOF: Multi-organ failure
ICU: Intensive care unit
ML: Machine learning
DT: Decision tree
SVM: Support vector machine
MLP: Multilayer perceptron
KNN: K-nearest neighbors
HOAs: Horse herd optimization
AI: Artificial intelligence
LOS: Length of stay
ALT: Alanine aminotransferase
ASP: Aspartate aminotransferase
ESR: Erythrocyte sedimentation rate
CRP: C-reactive protein
PSO: Particle swarm optimization
GA: Genetic algorithm
GWO: Grey wolf optimization
DE: Differential evolution
KNN: K-nearest neighbors
ANNs: Artificial neural

## Declarations

### Availability of data and material

All data generated and analyzed during the current study are not publicly available but are available from the corresponding author upon reasonable request and the Abadan University of Medical Sciences’ approval.
